# Evaluation of serum 25-Hydroxy vitamin D levels in children with autism Spectrum disorder

**DOI:** 10.1186/s13052-018-0587-5

**Published:** 2018-12-17

**Authors:** Ali Asghar Arastoo, Hesam Khojastehkia, Zahra Rahimi, Morteza Abdullatif Khafaie, Syed Ahmad Hosseini, Syed Mohammadtaghi Mansoori, Shabnam Yosefyshad, Maryam Abshirini, Noshin Karimimalekabadi, Maria Cheraghi

**Affiliations:** 1 Social Determinant of Health Research Center, Ahvaz Jundishapur University of Medical Sciences, Ahvaz, Iran; 2 Department of Public Health, Health School, Ahvaz Jundishapur University of Medical Sciences, Ahvaz, Iran; 3 Cherraan’s college of pharmacy Dr. MGR Medical University, Chennai, India; 4 Ahvaz Jundishapur University of Medical Sciences, Ahvaz, Iran; 5 Department of Nutrition, School of Para Medicine, Ahvaz Jundishapur University of Medical Sciences, Ahvaz, Iran; 6 School of Pharmacology, Mashhad University of Medical Sciences, Mashhad, Iran

**Keywords:** Autism, Iran, Vitamin D

## Abstract

**Background:**

Vitamin D plays an important role in etiology of Autism Spectrum Disorders (ASDs). We aimed to evaluate the serum 25 - hydroxyl vitamin D level among children with ASDs in Ahvaz city, Iran.

**Methods:**

It was a cross-sectional study which had conducted on 62 subjects in two groups: a case group (*n* = 31) consisted of ASD children who study in especial schools; and a control group (*n* = 31) of healthy children who were selected by simple random sampling from regular schools in Ahvaz city, Iran during 2016. Maching between two groups has done regarding Socioeconomic status, type and amount of food intake, place of living and age. The levels of serum 25 - hydroxyl vitamin D were assessed in early morning means fasted state and also measured using ELISA method. Data were analyzed using Statistical Package for Social Sciences (SPSS) version 20. The significant level was considered < 0.05.

**Results:**

In ASD children, the average serum 25-hydroxyvitamine D level was 9.03 ± 4.14 ng/mg. In ASD group, 96.8% (30 subjects) had vitamin D deficiency. In healthy children group, average serum 25-hydroxyvitamine D level was 15.25 ± 7.89 ng/mg. Average serum 25-hydroxyvitamine D level in intervention group was significantly lower than the control group (*P* > 0.001). Although the parents of patients in control group reported longer exposure to sun (27.42 m per day against 33.06 m per day), no significant difference was observed between these groups in terms of exposure to sun (*P* < 0.05).

**Conclusions:**

A significant difference was observed between serum 25-hydroxyvitamine D levels between the healthy and ASD children. It is recommended to use vitamin D supplement in children with ASDs under medical care.

## Introduction

Vitamin D may play an important role in etiology of Autism Spectrum Disorders (ASDs). Vitamin D is a neuroactive steroid affecting brain development and function. It plays an essential role in myelination, which is important for connectivity in the brain. Studies have shown that decreased vitamin D levels, decreased maternal vitamin D levels during pregnancy, and decreased exposure to solar UVB might increase the risk of ASD [1].

Despite extensive studies on ASD, the etiology of this disorder is quite unknown and studies are ongoing [2, 3]. ASD has a dominant genetic origin. However, environmental and genetic factors have interaction in the incidence of this disorder [3–5]. The results of studies have shown that in disease etiology, risk factors such as prenatal and postnatal infections [6, 7], and exposure to Valproic Acid of alcohol during pregnancy [8, 9], the age of mother [10], and abnormal nutritional and metabolic factors [3] are effective. During the recent years, the incidence of ADSs has been significantly increased. In previous studies, the incidence of this disorder was 10 in 10,000 [11], whereas the incidence of this disorder is now estimated as 90–250 in 10,000 [12–15]. In addition, in 2010, CDC reported the incidence of autism disorder in the United States as 1 in 68 and this indicates 78% increase in the incidence level compared with 2002 [16]. However, a part of this sudden increase is probably the result of increased awareness and better reports about autism disorder as well as improved diagnostic criteria, but the exact causes for this sudden increase should be determined in future studies [17]. Increased incidence of the disease can impose a heave financial burden on the society. It is estimated that medication costs for each patient will be 40,000 to 60,000 dollar per year [16]. During the past decades, numerous studies were conducted on the role of vitamin D in neuropsychological disorders [18–23]. The findings of these studies showed that vitamin D deficiency is one of the risk factors of evolutional neuropsychological disorders such as schizophrenia [24] and autism [19, 25–28]. However, studies on the relationship between vitamin D and autism in different parts of the world such as Sweden [29], Egypt [20], Saudi Arabia [30], and China [31, 32] indicate lower 25 (OH) D level in patients with ASD in different ages compared with the control group. Moreover, some studies [33, 34] have shown different findings and no significant difference was observed between serum levels of vitamin D in ADS and control groups. To our knowledge few studies have been conducted in this regard in Iran and no study has been conducted in Ahvaz city, Southwestern Iran. Therefore, the present study aimed to evaluate the serum 25 - hydroxyl vitamin D level among children with ASDs in Ahvaz city, Iran.

## Methods

It was a cross-sectional study which had conducted on 62 children in two groups: a case group (*n* = 31) consisted of ASD children who study in especial schools; and a control group (*n* = 31) of healthy children were selected from regular schools by using simple random sampling approach in Ahvaz city, Iran; 2016.

The two groups were matched in terms of gender, age, weight, height, head circumference, adequate breastfeeding (for at least six months), type and amount of food, socioeconomic status (the ratio of the number of family members to bedrooms was used as a measure of socioeconomic status) [35, 36], average income, family size, and exposure to smokers.

### Inclusion criteria

Students with ASDs entered the study by confirming the diagnosis by a neurologist and based on DSM-IV criteria and obtaining written informed consent from the parents. In the control group, the informed consent of parents was among the inclusion criteria, too.

**Exclusion Criteria** of the study were the presence of epilepsy and the use of vitamin D supplements were considered as exclusion criteria.

### Clinical evaluation of patients with autism

Diagnosis of patients with ASD based on medical experience, clinical examination, and two criteria of DSM-IV and ADI-R was confirmed by a neurology expert.

### Evaluation of serum 25-hydroxyvitamine D level

The levels of serum 25 - hydroxyl vitamin D were assessed in early morning means fasted state.

An expert nurse collected 5 mL blood of children to measure 25 (OH) D serum levels in a Blood Transfusion Center in Ahvaz city, Khuzestan, Iran. The serum samples were isolated after centrifugation and kept at − 20 °C until the laboratory assessments. The serum 25 (OH) D levels were measured using the ELISA method (Euroimmun kit, Medizinische Labordiagnostika AG, Germany, EQ. 6411–9601).

According to the guidelines of the American Endocrine Association, vitamin D level is defined with the concentration of 25-hydroxyvitamine D3 in blood. Natural, insufficient, and deficient vitamin D levels were determined with 25-hydroxyvitamine D level lower than 20 ng/ml, 21–29 ng/ml, and 30 ng/ml, respectively [36].

All the tests in this study were performed in Nargess Laboratory in Ahvaz city under the supervision of a doctor of medical laboratory sciences. Tests were performed two times and the averaged values were used in the analyses to increase the accuracy of the results.

### Ethical considerations

Prior permissions from educational authorities, school principals, and class teachers were obtained, and then written informed consent form was taken from the parents’ children participate. The procedures of this study were approved by Independence Ethics Committee of Ahvaz Jundishapur University of Medical Sciences, Ahvaz, Iran (IR.AJUMS.REC.1394.199) also we have thanks to all subjects and their parents to participate in this study. Parents of children had informed consent to participate in this study.

### Statistical analysis

Kolmogorov-Smirnov test was performed prior to statistical analysis to examine the normality of the variables. The results were presented in the form of statistical tables and numeric indicators. Chi-square test, t-test, and nonparametric test (Mann-Whitney U test) were used to analyze the data. Variable values were expressed as frequency, mean ± standard deviation (SD). Statistical calculations were performed using Statistical Package for Social Sciences version 20 (SPSS Inc., Chicago, IL, USA). For all statistical analyses, *P* value less than 0.05 was considered as significant.

## Results

The finding has shown that 31 children with ASD and 31 healthy children with age range of 5–12 years old. No significant difference was observed between two groups in terms of age (*P* = 0.80). In addition, no significant difference was observed between the mean of mother’s age at the birth of the child in both groups (*P* = 0.28). Most of the subjects in the case and control groups were male (83.9 and 90.3%, respectively) and no significant difference was observed in both groups in terms of gender (*P* = 0.45). Most of children in the case group were Bakhtiari (35.5%) and in the control group were Arab (Table 1).

**Table 1:** Demographic characteristics in two groups

Variable	ASD N (%)	Healthy children Number (%)	*P*-value
Gender			
Male	26 (83.9)	28 (90.3)	0.45^a^
Female	5 (16.1)	3 (9.7)	
Father’s ethnicity			
Fars	6 (19.4)	6 (19.4)	0.51^a^
Bakhtiari	11 (35.5)	7 (22.6)	
Arab	10 (32.3)	13 (41.9)	
Lor	1 (3.2)	3 (9.7)	
Tork	2 (6.5)	1 (3.2)	
Other	1 (3.2)	1 (3.2)	
	ASD Mean (SD)	Healthy children Mean (SD)	
Age	9.17 (2.11)	9.31 (2.09)	0.80^b^
Height	142.35 (14.23)	137.06 (12.52)	0.12^b^
Weight	42.37 (19.55)	36.13 (12.53)	0.14^b^
Age of mother at Childbirth	35.52 (10.90)	33.22 (4.60)	0.28^b^

^a^Chi-square test ^b^Mann-Whitney test

**Table 2:** Serum vitamin D level and exposure to direct sun in two groups

Variable	ASD Mean (SD)	Healthy children Mean (SD)	*P*-Value
serum 25-hydroxyvitamine D level ng/mg	9.03 (4.14)	15.25 (7.89)	< 0.001
Time of exposure to sun	27.42 (33.14)	33.06 (42.94)	0.56
	ASD N (%)	Healthy children N (%)	
Deficient of Vitamin D (≤20 ng/ml)	30 (96.77)	22 (70.97)	0.006
Insufficient of Vitamin D (21–29 ng/ml)	1 (3.22)	9 (29.03)	

In ASD children, the serum 25-hydroxy vitamin D level was significantly lower than the control group (*P* > 0.001). In the ASD group, all children showed deficient or insufficient level of serum 25-hydroxy vitamin D (96.8% or 3.2%, respectively).

In both groups, the use of direct daily sun was equal (67.74%). No significant difference was observed between the groups in exposure to direct sun (min/day) (P-0.56) (Table 2).

## Discussion

Recently, the role of vitamin D deficiency is identified as an environmental risk factor for some of autoimmune disorders [37, 38]. A study by Patrick and colleagues showed that vitamin D ma influence some of social behaviors of children with autism. He emphasized that vitamin D is a gene activator that creates tryptophan hydroxylase enzyme. This enzyme converts tryptophan into serotonin in the brain. Therefore, a sufficient level of vitamin D to produce serotonin in the brain than functions as a neuron transmitter improves social behaviors by positive effects on behavior [39]. In a clinical trial study by Feng and colleagues on 37 children with autism, for three months, these children received 150,000 IU as intramuscular injection(monthly) and 400 IU orally (daily). These researchers reported that disease symptoms and behavioral checklist in children (3 years old and older) with autism improved [40]. In most of the studies conducted on ASDs and vitamin D, lower 25-hydroxyvitamine D level in children with autism was taken into consideration. Our findings showed low level 25-hydroxyvitamine D level in the ASD children, compared with the healthy counterparts. Testes et al. reported that the mean of serum 25-hydroxyvitamine D level in children with autism with different ethnicities was lower than the control group by 35 nmol/L [41]. Moreover, Duan et al. showed that serum 25-hydroxyvitamine D in patients with autism was significantly lower than the control group [31]. Bener et al. attributed some biological and lifestyle factors such as birth, kinship, body mass profile, and physical activity and 25-hydroxyvitamine D level with the incidence of autism. Their findings showed that serum 25-hydroxyvitamine D level in ASD children was lower than the control group with similar ethnicity, age, and gender (*P* = 0.004) [42]. This finding was supported by the study of Meguid et al. [20]. Saad et al. [18] reported an inverse relationship between the averaged serum 25-hydroxyvitamine D level and severity of ASD (*P* > 0.001), which was not evaluated in our study. In another study in Saudi Arabia on comparing serum 25-hydroxyvitamine D level and MAG among 50 children with autism (5–12 years old) and 30 healthy children, a significant negative relationship was observed between serum 25-hydroxyvitamine D level and incidence of autism (*P* > 0.001) [30]. Neumeyer and colleagues reported that the ration of male children with ASD with serum 25-hydroxyvitamine D level lower than 80 nmol/L was higher than healthy subjects (77% against 37% and *p* = 0.02). However, the results of a study by Molloy [33] and Esparham [3] in the United States showed that there is not significant relationship between serum 25-hydroxyvitamine D level in two groups of children with ASD and without ASD. Ugur and colleagues investigated vitamin D3 level of 54 children with autism and 54 healthy children between 3 and 8 years old in Turkey. They did not observe any significant difference t vitamin D3 serum level between these two groups [35]. The results of a study by Hashemzeh and colleagues in Iran showed no significant difference between vitamin D in children with autism and healthy children. Also, no significant relationship was observed between serum vitamin D level and severity of the disease symptoms [34].

## Conclusion

There significant difference was observed between serum 25-hydroxyvitamine D levels in two groups of this study and different studies confirm that and also there was no significant difference between two groups in time of exposure to sun. Therefore it is recommended to use vitamin D supplement in children with ASDs under medical care.
