# Correction to: Evaluation of serum 25-Hydroxy vitamin D levels in children with autism Spectrum disorder

**DOI:** 10.1186/s13052-019-0611-4

**Published:** 2019-02-04

**Authors:** Ali Asghar Arastoo, Hesam Khojastehkia, Zahra Rahimi, Morteza Abdullatif Khafaie, Syed Ahmad Hosseini, Mohammad Taghi Mansouri, Shabnam Yosefyshad, Maryam Abshirini, Karimimalekabadi Noshin, Maria Cheraghi

**Affiliations:** 1 Social Determinant of Health Research Center, Ahvaz Jundishapur University of Medical Sciences, Ahvaz, Iran; 2 Department of Public Health, Health School, Ahvaz Jundishapur University of Medical Sciences, Ahvaz, Iran; 3 Cherraan’s college of pharmacy Dr. MGR Medical University, Chennai, India; 4 Ahvaz Jundishapur University of Medical Sciences, Ahvaz, Iran; 5 Department of Nutrition, School of Para Medicine, Ahvaz Jundishapur University of Medical Sciences, Ahvaz, Iran; 6 Department of Anesthesiology, Columbia University Irving Medical Center, New York, NY 10032 USA

**Correction to: Ital J Pediatr**

**https://doi.org/10.1186/s13052-018-0587-5**

The original article [1] contained an error in author Mohammad Taghi Mansouri’s name. The correct spelling is now shown in the original article as well as this Correction article.
